# Congenital Adrenal Hyperplasia Presenting as Pulseless Ventricular Tachycardia in a Neonate

**DOI:** 10.7759/cureus.4749

**Published:** 2019-05-24

**Authors:** Nida Manzoor, Areeba Minhaj, Manahil Akmal

**Affiliations:** 1 Pediatrics, Civil Hospital Karachi, Karachi, PAK; 2 Internal Medicine, Dow University of Health Sciences, Karachi, PAK

**Keywords:** congenital adrenal hyperplasia, pulseless ventricular tachycardia

## Abstract

Congenital adrenal hyperplasia (CAH) comprises a group of autosomal recessive inherited disorders that arise due to defects in one of the enzymes of steroidogenesis pathway in the adrenal glands. Ninety-five percent of the cases occur due to deficiency in 21-hydroxylase (21-OH). Clinically, CAH due to 21-OH deficiency presents in two distinct forms, classic CAH and non-classic CAH. Females with classical forms present with genial ambiguity while the presentation in males is more subtle with severe electrolyte disturbances being the initial manifestation in many cases. Arrhythmias are a rare manifestation of CAH. We report the case of an 18-day-old male child who presented with pulseless ventricular tachycardia and was later diagnosed with congenital adrenal hyperplasia based on the laboratory findings of elevated 17-hydroxyprogesterone (17-OHP) levels. Our case reveals that fatal arrhythmias such as a pulseless ventricular tachycardia can be the primary manifestation of the adrenal insufficiency of CAH even in the absence of any physical findings and hence clinicians should always maintain a strong suspicion for CAH in any child presenting with unexplained arrhythmia. Furthermore, this case also highlights the need for CAH screening in neonates so that the appropriate hormone replacement can be initiated before the development of life-threatening adrenal crisis.

## Introduction

Congenital adrenal hyperplasia (CAH) comprises a group of autosomal recessive inherited disorders that arise due to defects in one of the enzymes of steroidogenesis pathway in the adrenal glands. More than 95% of cases of CAH are due to the deficiency of 21-hydroxylase, the enzyme responsible for the conversion of progesterone into deoxycorticosterone and 17-hydroxyprogesterone to 11-deoxycortisol [1-3]. This deficiency occurs due to mutations in CYP21A2 and results in decreased levels of cortisol and aldosterone on one hand and on the other hand, increased levels of adrenal androgens and precursors of steroids such as 17-hydroxyprogesterone (17-OHP) [4]. Decreased cortisol levels stimulate the release of adreno-corticotropic hormone (ACTH) from the pituitary gland leading to hyperplasia of the adrenal glands and a concomitant increase in synthesis of steroid precursors which are converted into sex hormones, particularly testosterone responsible for characteristic signs and symptoms of CAH [3]. Deficiency of aldosterone leads to electrolyte disturbances including hyponatremia, hyperkalemia, hypovolemia, shock which can ultimately result in death. Clinically, CAH due to 21-hydroxylase (21-OH) deficiency presents in two distinct forms, classical CAH and non-classical CAH. Classical CAH is further divided into simple virilizing (SV-CAH) and salt wasting (SW-CAH) forms [4]. In most populations, classic CAH has an incidence of 1:11,800 to 1:21,800 live births [5]. Classical CAH in females usually presents at birth with genital ambiguity due to high androgen levels in utero leading to genital virilization, whereas in males, in the absence of genital defects, salt loss at 7-14 days with hyponatremia, hyperkalemia, weight loss and lethargy may be the first sign of the disease [6]. Patients with non-classic CAH are usually asymptomatic or may present with precocious puberty, rapid weight gain and an advanced bone age.

In males with classic CAH, the electrolyte imbalances may present before the recognition of genital abnormalities. The subtle virilizing features in males make CAH difficult to diagnose until the electrolyte imbalance is severe.

Patients with CAH are prone to develop cardiac arrhythmias due to electrolyte imbalances particularly due to hyperkalemia. Some rare cases of CAH have presented in the form of cardiac arrest [7] and ventricular tachycardia [8]. Here we present an intriguing case of a 20-day-old neonate presenting with pulseless ventricular tachycardia and laboratory evidence of CAH with no other physical findings consistent with CAH. To our knowledge this is the only reported case of CAH in which the first documented rhythm is pulseless ventricular tachycardia.

## Case presentation

An 18-day-old male baby, first product of a non-consanguineous marriage, born at full term through normal vaginal delivery was brought to the emergency department in an unresponsive state. According to the parents, the child had been vomiting and eating poorly for the past two days. Birth history was unremarkable with no antenatal and postnatal complications.

On admission, his blood pressure and peripheral pulses were undetectable. He was bradycardiac (heart rate 40/minute) and moderately dehydrated. He was unresponsive with shallow breathing the respiratory rate being 33 breaths per minute. His oxygen saturation was 92% and the temperature was 37°C. Capillary refill time was found to be four seconds. Random blood sugar came out to be 32 mg/dl. Cardiovascular examination revealed muffled heart sounds but no murmurs. Central nervous system examination revealed normal tone, reactive pupils and normal fontanelles. The remainder of the systemic examination was also unremarkable.

**Figure 1:**
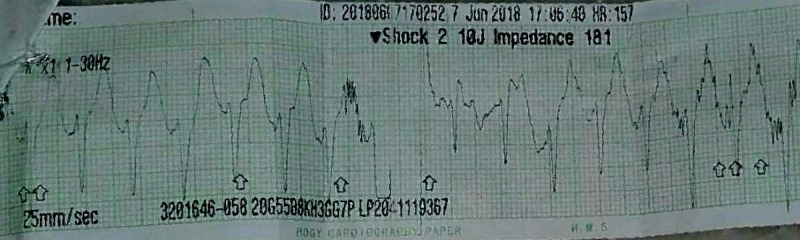
Electrocardiogram tracing showing ventricular tachycardia with absent P waves and broad QRS complexes

An electrocardiogram (ECG) was instantly obtained which revealed ventricular tachycardia (Figure 1). Other ECG findings included absent P waves and wide QRS complexes. Airbag and mask ventilation was started along with cardiopulmonary resuscitation. Epinephrine was injected at dose 0.01 mg/kg IV stat while call for cardioversion was given. The baby was cardioverted twice and IV amiodarone at 5 mg/kg loading dose was commenced, after which the normal sinus rhythm was obtained and the baby started responding. His heart rate increased up to 150 beats/min, respiratory rate was now 48 breaths/minute, blood pressure 62/37 mm of Hg, oxygen saturation was 99%. He was then put on IV amiodarone and IV epinephrine infusions and shifted to neonatal intensive care unit (NICU).

Laboratory test results showed hemoglobin 15.1 g/dl, mean cell volume 90 fL, total leukocyte count 26,600/mm^3^ with neutrophils 56.3%, lymphocytes 31.8%, platelets 652,000/mm^3^ and C-reactive protein (CRP) 1.9.

Electrolyte report revealed sodium 123 mEq/dl, potassium 6.0 mEq/dl, chloride 80 mEq/dl, calcium 9.6 mg/dl and magnesium 1.9 mg/dl while creatinine and blood urea nitrogen (BUN) were 1.4 mg/dl and 37 mg/dl, respectively.

His labs revealed significant hyperkalemia which was most likely the underlying cause of ventricular tachycardia. The high potassium and low sodium levels made congenital adrenal hyperplasia a plausible diagnosis.

A laboratory test for the detection of 17-hydroxyprogesterone was sent. The test revealed high levels of 17-hydroxyprogesterone (320 ng/ml). Renal ultrasound was done to check for adrenal hyperplasia but it came out to be normal. The genital examination was unremarkable with no ambiguity, the penis was of normal length and no skin hyperpigmentation was noted on the axilla, neck, and genitals. The child was diagnosed with CAH based on the laboratory results of increased levels of 17-hydroxyprogesterone. To determine the type of CAH, tests for plasma renin and aldosterone were also performed. Plasma renin came out to be elevated (>500 uIU/ml; normal in supine position 2.3-39.9 uIU/ml and in erect position 4.4-46.1 uIU/ml). Serum aldosterone was also high (15.40 ng/dl; normal in recumbent position 1.5-13.3 ng/dl).

The infant was discharged on hydrocortisone (15 mg/m^2^/24 hours in three divided doses) and fludrocortisone (0.2 mg daily in two divided doses) along with supplementary NaCl (8 mmol/kg). Parents were advised to consult the doctor in case the child fell ill, as stress requires increment in the dose of glucocorticoids to prevent adrenal crisis. The dose was also increased prior to circumcision. Follow-ups in the outpatient department have shown normal electrolytes and ECG and optimal growth and development of the child.

## Discussion

Classic CAH due to 21-hydroxylase deficiency presents in two forms: salt-wasting form and simple virilizing form. The affected newborns in the salt-wasting form present with life-threatening adrenal crisis a few days after birth due to inadequate aldosterone secretion. Salt-wasting form is characterized by low serum aldosterone and cortisol and increased plasma renin activity (PRA). The simple virilizing form has no features of salt-losing crisis and usually manifests late in childhood with precocious puberty or with clitoral or penile enlargement secondary to excess adrenal androgens [9].

Our patient presented with life-threatening salt crisis and electrolyte imbalances, features of classic CAH-salt wasting form. Patients with classical CAH are prone to develop cardiac arrhythmias secondary to electrolyte imbalances particularly due to hyperkalemia. However, in most cases of CAH, hyperkalemia resulted in sudden cardiac death and arrhythmias have rarely been documented [7].

Hyperkalemia is defined as a potassium level of >5.5 mEq/l. The ECG findings of hyperkalemia are progressive from tall peaked T waves, a shortened QT interval to prolongation of PR interval and loss of P waves and then to diffuse widening of QRS complex and if untreated, death [10].

Shockable rhythms, like pulseless ventricular tachycardia or PVT, are a rare event in neonates [11-13]. The ECG in ventricular tachycardia shows wide QRS complex, often unidentifiable P waves, but when present, opposite polarity T waves, and AV dissociation are characteristic. The ventricular rate is regular and at least 120/min. Heart disease, prolonged QT syndrome, cardiomyopathy, and electrolyte imbalances such as hyperkalemia, are some of the causes of ventricular tachycardia. In our case, however, the underlying cause of PVT was found to be CAH.

Deterioration of VT into pulseless ventricular tachycardia and ventricular fibrillation may occur. In pulseless VT, the patient is apneic, unresponsive with undetectable peripheral pulses, as was documented in our case. The management of ventricular tachycardia and pulseless ventricular tachycardia is different. Pulseless VT is a shockable rhythm and early recognition is necessary for prompt management [14] as was successfully done in this case.

A similar case of ventricular tachycardia in a 20-day-old male child with CAH was reported by Virdi et al. [8]. The correction of electrolyte abnormalities, in this case, restored the normal sinus rhythm. Diagnosis of CAH was based on elevated 17-OHP levels (59.0 ng/ml; normal range- not detectable to 0.60 ng/ml) and evidence of genital virilization (increased penile length and hyperpigmentation of the external genitalia). Agarwal et al. document a case of sudden cardiac arrest in a child who was diagnosed to have CAH based on markedly increased 17-OHP levels (351 ng/ml) and evidence of adrenal hyperplasia on ultrasound [7].

Salt-wasting form of classical CAH is characterized by low levels of serum aldosterone [9]. Surprisingly, the aldosterone levels in our case were normal. This can be due to the fact that aldosterone levels were sent for five days after the treatment was initiated which could have altered the results. Another possible explanation of normal aldosterone levels in a CAH child with salt wasting is the cross-reactivity of steroid precursors which spuriously elevates aldosterone levels in aldosterone assay as documented by Tuhan et al. [15] and reported by Boddu and Madhavan [16].

The adrenal ultrasound of this child was also unexpectedly normal; although unusual, this does not rule out the diagnosis of CAH and normal adrenal ultrasound in untreated children with CAH has been documented by Al-Alwan et al. [17].

21-hydroxylase deficiency, in its classic SW form, is characterized by markedly elevated levels of 17-OHP usually >600 nmol/ml or >2000 ng/ml. Corticosteroids are the mainstay of treatment of classical CAH [9]. Treatment comprises of glucocorticoids, mineralocorticoids and salt supplements to maintain plasma sodium concentration and renin within the normal range.

Newborn screening programs using 17-OHP assays have been initiated in many countries resulting in a considerable decrease in morbidity and mortality that was previously attributable to the severe adrenal crisis in salt-wasting forms of CAH [9]. Reviews from Europe show that none of the neonates who were diagnosed with CAH based on screening assays had clinical findings suggestive of the disorder [18]. However, in developing countries like Pakistan, in the absence of any screening program, life-threatening arrhythmias and severe salt-losing crisis can be the only initial manifestation of CAH and thus clinicians should maintain a strong suspicion of CAH in all the infants with unexplained arrhythmias.

## Conclusions

Our case reveals that rare fatal arrhythmias, such as a pulseless ventricular tachycardia, can be the primary manifestation of the adrenal insufficiency of CAH in a neonate even in the absence of any physical findings. Electrolyte testing should be sought in any infant who presents with significant unexplained arrhythmias without any evidence of congenital heart disease. Moreover, our case also highlights the need for CAH screening in newborns in developing countries like Pakistan so that affected neonates can be identified before the development of the life-threatening salt-losing crisis and appropriate hormone replacement be initiated.
